# Gynecological Mistakes and Their Pathological Bearing and Importance1Read before the Section on Pathology, Buffalo Academy of Medicine, April 18, 1900.

**Published:** 1900-07

**Authors:** H. E. Hayd

**Affiliations:** Buffalo, N. Y., Gynecologist to the German Deaconess’s Hospital


### BUFFALO MEDICAL JOURNAL.

VOL. XXXIX.—LV. JULY, 1900. No. 12.

### Original Communications.

### GYNECOLOGICAL MISTAKES AND THEIR PATHOLOGICAL BEARING AND IMPORTANCE.[1]

By H. E. HAYD, M. D., Buffalo, N. Y.,

Gynecologist to the German Deaconess's Hospital.

IN THE consideration of the pathology of pelvic disease, it must be accepted that inflammatory affections of the endometrium, tubes, ovaries and peritoneum, are the result of the same pyogenic organisms as produce inflammations elsewhere, and just as we have mild and grave clinical manifestations in the cellular tissues of the extremities as the result of this coccic invasion, so we see different degrees and forms of pelvic trouble; the staphylococcus producing the milder forms of endometritis, salpingitis, ovaritis and peritonitis, while the streptococcus is responsible for the more virulent forms as well as the broad ligament phlegmons, ovarian abscess, purulent peritonitis and metastatic involvement of joints and distant organs. Besides those and perhaps other pyogenic bacteria, the gonococcus plays a most important rôle, and is perhaps responsible for as much, if not more, pelvic disease than all the other forms put together. Usually it does not exist alone, but in conjunction with the other pus-producing agencies, making a mixed infection and capable of causing the most serious complications the surgeon is ever called upon to encounter, imperiling not only the life of the patient, but destroying the function and usefulness of the organs involved.

These different cocci enter the cervix and occasion an acute endocervicitis, or they may remain there in a more or less latent fashion and come into pernicious activity by various traumatic or other agencies. The gonococcus travels by continuity of tissue to the endometrium and thence to the tubes and ovaries and peritoneum, while

1. Read before the Section on Pathology, Buffalo Academy of Medicine, April 18, 1900.

the other pus-producing bacteria, the staphylococcus and streptococcus, may also take the course of the lymph spaces and veins, directly to the peritoneum, and involve ovaries and tubes secondarily. Sometimes, although rarely, the intestinal tract is the source of the infection, by reason of the bacterium coli communi, which passes through the intestinal wall, and occasionally the gonococcus enters the peritoneum through the bladder or ureters and kidney.

However it must not be understood that all the diseases of the female generative tract are the result of bacterial invasion, as it is very probable that the catarrhal diseases of young girls and virgin women, with their associated creamy leucorrheas, are of purely hematogenous origin and are only made serious by reason of unnecessary examinations and local manipulations with sounds and intra-uterine applicators, converting a simple catarrh into a purulent inflammation. Nor can we accept in the light of recent pathology, cold feet, frights, emotions and disturbances of menstruation as a cause for pus in the pelvis, as these are but excuses for dirty finger nails, unclean surgical instruments and infidelity.

It would be extremely interesting for us to follow the pathological appearances of the organs involved in these diseased processes, but I purposely leave this study, feeling that a more practical and useful paper will result from the clinical application of these great under-mining principles of the causes of pelvic disease. By this knowledge and a scientific respect for these deductions, we can at once see the dangers in connection with the therapeutics of the older school of practitioners, who believed and advocated that the uterine cavity could be invaded with sounds and syringes and various local treatments made without any special danger or harm.

However, with our new pathology, came increased wisdom and with it the greatest respect for the vulnerability of the endometrial mucous membrane, a knowledge which teaches us that although it may suffer all kinds of abuses without any serious reactions sometimes, yet sooner or later, the most serious consequences will result from the most trivial injury. The symptomatology of uterine disease is complex and many-sided, some of its symptoms are manifested in the organ itself, while others partake of the most diversified reflex phenomena. However, upon careful inquiry, certain prominent symptoms often suggest themselves, which point strongly to certain definite lesions, as for instance, the hemorrhage in fibroid tumor, the dysmenorrhea in endometrial affections of the cervix and body of the uterus, the reflex symptoms and often to most remote organs, from displacements. The uterine syndroma of Pozzi, embraces certain general symptoms which accompany pelvic diseases and may be said to be pain, leucorrhea, dysmenorrhea, metrorrhagia, symptoms from neighboring organs, as the bladder and rectum, and from distant organs as the stomach and digestive tract, and the nervous system generally. Unfortunately many of these symptoms are present in other conditions than true uterine, tubal or ovarian disease and therefore their sources must be sought and if possible understood and carefully studied out.

Women, as a class, assume that most of their distress, pain and suffering originates in, or results from their genital organs, and look upon their sex as a providential curse. They are, therefore, always ready to believe, with but little encouragement, that some special disorder exists, and thus they become the victims of unnecessary and painful examinations and much needless local treatment, or as Price would say "gynecological tinkering." Pain in the ovarian region, dysmenorrhea, leucorrhea and backache are often but the expression of a poorly nourished body and an unbalanced nervous system, worn out by excessive cares and anxieties, and driven to exhaustion by unrequited affection and domestic infelicity. Medicine being an inexact science, commands many agencies potent for good and often fraught with harm, and these are placed into the hands of careless, unqualified and often unscrupulous medical men, and as a result, simple functional disorders are converted into gross lesions which only skilful and desperate surgical procedures can remedy. Pain is no doubt the most frequent symptom which we are called upon to treat, and is the one which most readily begets our sympathy, and should have our best professional thought. It is a symptom of so many varied conditions, and is so complex in its origin, being often most acute in purely functional disturbances, that it is frequently assumed to be the expression of some serious lesion, and as a result, many needless mutilating operations have been vainly tried for its relief. Lomer, of Hamburg, has beautifully studied this question, and has written two most valuable articles, which are to be found in the April and May (1899) numbers of the *Journal of Obstetrics and Gynecology*, and deserve your most careful study. Many women have been operated upon for the relief of pain, in whom gross painful lesions existed, and yet were not relieved of their pain by the operation, because there was also present, a reflex pain and tenderness, or a brain pain, which may have been due to the presence of the growth or the inflammatory exudate, or may have been independent of them altogether. As a rule, however, a nervous or hysterical pain is acute, and is attended with marked and perhaps excessive superficial tenderness. It is in the skin and can be often increased by raising the skin into folds, and it is unduly disproportionate to the diseased organ. There are also present painful stigmata elsewhere and other well-marked hysterical manifestations, as the globus and the anesthetic conjunctiva and soft palate. It is needless for me to allude to the many operations which have been performed for the relief of pain, and how many querulous and mutilated women are going about from doctor to doctor, seeking relief for the many diverse and horrible symptoms which have resulted from an improper estimation and appreciation of this subtle but coy symptom, pain; suffice it to say, that these operations, which are more often performed by the unwary and the unguarded, are justly the opprobium of our surgical art.

Endometritis and cervical catarrh is a very common affection with women, and is so easily influenced by conditions of the general health, that most women during their active menstrual life, suffer from them. They are the simple diseases of the uterus that we are so often called upon to prescribe for and they are the slighter ailments which men who hardly know the use of a speculum, institute active treatment against. Here the various caustics are applied, from the simple nitrate of silver solutions to the fuming nitric acid. Stenosis of the cervix and os interum and even cicatricial contraction of the canal, frequently results from these topical applications, as well as acute infectious endometritis with tubal and ovarian complications, often by lighting up latent gonococci and other pus-producing bacteria. Tonics, good food, change of air, active exercise, daily movement of the bowels, sexual rest and astringent injections are usually all that is called for. If the case, however, proves intractable and rebellious, a properly performed curettage and packing with iodoform gauze, is an infinitely less dangerous and far more effective means of treatment. However, if the cervix is wide and patulous and admits of free drainage, and there is no associated tubal or ovarian mischief, intrauterine applications of iodine or iodine and carbolic acid, with proper precautions, may be safely employed; but if the internal os is small and contracted, much pain and colic will result, and perhaps tubal complications. It is absurd to assume, except in the most exceptional cases, that a uterus can be properly curetted, without the assistance of an anesthetic, and that the operation can with safety be done in one's office and the patient be permitted to go home, as after an ordinary intrauterine treatment. I am satisfied that great harm is done women every day by otherwise capable men, by underestimating the importance of this truly surgical procedure. It cannot be done well, and it will not be done thoroughly and cleanly, in a patient who is not anesthetised. Unfortunately, this splendid operation, capable of so much good when indicated, is performed quite unnecessarily, because mechanically it is so easily done. Here the merest tyro in medicine, in order to be in the swim with his successful rival and teacher, who is an acknowledged operator of ability, curettes, because he is not capable of doing the indicated Alexander operation, or an abdominal section. He persuades his poor, guileless and trusting patient that a curettage will make a speedy cure, and thus gets the credit of doing an operation. This experience, I have had time without number, and have taken out big pus tubes and ovaries in women, in whom one month before, a curettage was performed. The mechanical side of surgery is but a small matter and difficult technique is soon studied, but to be a diagnostician requires patience, study, practice and great skill, and without this ability to diagnosticate surgical conditions, surgery must be robbed of its glory and usefulness.

Salpingitis and ovaritis are often associated with the endometritis; in fact it is questionable whether an endometritis pure and simple exists alone, since the contiguity of similar mucous membranes, offers the route of an ascending inflammation which may be of a very mild degree in the tubes, and evidenced clinically only by slight tenderness without fullness in the vaginal fornices, but by the slightest provocation, may be accentuated so that the tube gets swollen, and its fimbriated end turns in and closes, when we get retention cysts, hydrosalpinx and pyosalpinx diseases which practically disorganise the future usefulness of the channel, or later pachysalpingitis with its sclerosing changes, making conservative surgery upon the tubes without hope. These are serious matters for the future well-being of woman, as well as the perpetuity of the race and nation.

Cervical lacerations with their associated erosions, or what are commonly called ulcers, are often shamefully treated by capable and representative men in our profession. They are painted with tincture of iodine and various caustic preparations week in and week out, and when the patient is tired out by these frequent applications without relief, she of her own volition, seeks a surgeon who quickly performs a trachelorrhaphy and restores her to a healthy and useful life; or from neglect to inform the poor woman of her future possibilities and dangers, these unhealthy ulcerated tears go on slowly but often surely to malignant disease. Still unmindful and careless of our terrible responsibilities, this poor, trusting and confiding patient, who once had a simple and easily cured laceration, now has a cancerous ulceration, so far advanced and with infiltration so general into the broad ligament, that an operation, even if performed, is of questionable value. This gruesome picture looks like a creation of a vivid imagination, but yet these experiences, every one of us operating gynecologists have had, and these patients have been tamponned and painted with caustics inside of the womb and outside for weeks and months. It does not seem possible with the enlightenment of the nineteenth century, that such criminal ignorance and negligence could exist, but our operating rooms prove the truth and justice of my criticism. Neglected perineal lacerations, with their associated rectoceles and cystoceles, are treated by faradisation and alum injections and all kinds of tinkering and unscientific devices, until they become such serious matters that even well-directed plastic surgery cannot restore them to normal conditions.

Ureteritis, a quite common malady in women, is also carelessly interpreted, and its symptoms are treated as if they were of intrauterine origin, and by the same routine procedures which characterised the average gynecological outfit—speculum, sound and intrauterine applicators.

Diseases of the rectum have such a varied symptomatology that they are frequently unsuspected. Fissure of the anus will produce the most distressing backache and reflex symptoms even to remote organs, and occasion such a degree of ill-health and nervous unbalance as to be responsible for the most violent forms of neuralgia and congestive dysmenorrhea. I dilated and curetted a young woman once without relief for marked congestive dysmenorrhea, and subsequently found an unsuspected fissure of the rectum, not so painful or irritable as to have produced pain at stool, but sufficient to have caused such an amount of nervous leak as to have undermined her whole system and reflexly to have congested her pelvic organs. Upon dilating the sphincter thoroughly, she was cured of her painful menstruation. Our orificialists will maintain that her condition was due to a contracted sphincter, but be that as it may, her rectum was responsible for her pelvic mischief.

Nervous ovaries or Charcot's ovaries is also a very common condition, and is so often misunderstood and treated as though an organic affection existed. Pain in the ovarian regions and often excruciating tenderness in the iliac fossæ, with epigastric pain and distress, and sometimes marked vomiting and dysmenorrhea are its principal symptoms; while upon vaginal examination, the uterus is found to be freely movable and the lateral sulci not thickened or indurated, but often excessively tender. Here the fine faradic coil is a specific, and as if by magic, the pain and tenderness disappear and sometimes only one or two seances are necessary.

The uterine sound, once so frequently used for diagnostic purposes, has been replaced by the educated finger and has justly been relegated to a very small field in the gynecological armamentarium. Yet it is a two-edged sword, and although sometimes a very valuable instrument, should only be used with the greatest care and with the strictest antiseptic precautions. The same might be said in reference to all intrauterine applicators, and to all kinds of intrauterine manipulations.

It has been proven by frequent culture experiments that gonococci often remain latent for months and even years in the cervical canal and deep in the cervical mucous membrane, and are only brought into pernicious activity by the slight traumas produced by careless and unnecessary sounding and instrumentation of the uterus. In 100 consecutive women examined by Pryor, in twenty-four, the gonococcus was found, and perhaps was producing no trouble and only required the careless examination to be responsible for an acute infectious inflammation. The same clinical application can be made in reference to benign catarrhs which are insulted by the unclean sound or the contagiums of the vagina, which stick to it and thus more or less severe septic processes may follow.

The galvanic electrode has done more harm in the hands of the unskilful, than all the good which has been accomplished by its brilliant apostle, Apostoli, and his trained followers. And I regret to be compelled to say that so valuable an agent as electricity has been so easily placed in the hands of the unqualified and unscrupulous, that it is now being used by them, largely in our own city, under the guise of giving local treatments, for the purposes of criminal abortion.

The pessary, which is a troublesome and sometimes abominable device, under the most favorable circumstances, is yet a very useful instrument and capable in the hands of experienced men, of bringing great comfort and relief to suffering women. Every retrodisplacement, however does not produce definite symptoms, and perhaps does, only when there is present associated endometritis and catarrhal salpingitis. Nevertheless, it is too often the practice to insert this mechanical device where support is quite unnecessary, and when the symptomatology is due to some other totally remote condition. The dangers associated with the wearing of pessaries, when neglected and not properly taken care of, are too familiar to you all to need any further comment from me. You have all seen the incrustations and the ulcerations of the vaginal wall, from its careless and neglected application, and have also known of metritis, tubal and ovarian diseases to result from their prolonged use. The last decade has placed in our hands, the Alexander operation, a unique and ideal surgical procedure, for the relief of symptom-producing uterine retrodisplacements. A simple, bloodless and easily performed operation, and I should say, practically without danger in careful and experienced hands. An operation which does away with the necessity of wearing a more or less dangerous, and at all times a nasty, and often stinking mechanical device, and one where the future child-bearing function is not in any way endangered or interfered with. It must be performed, however, only in suitable cases—in simple, uncomplicated posterior displacements.

The cry is—and a just one too—that there is altogether too much operating done nowadays; yet in the face of this just criticism I am compelled to believe that most of the symptom-producing definite and discoverable lesions of women, are cured best and quickest by operative measures, and our work in the past was often too incomplete to get perfect results, because surgical means were not pushed far enough to cure all the conditions which were present. Recent pathology has shown us, and clinical experience has demonstrated that often with marked retroversions, there is a general enteroptosis, an associated floating kidney, and a resulting chronic appendicitis. To place the uterus in position is but one element in relieving symptoms and distress, and without fixing the kidney and perhaps removing the appendix, a cure cannot possibly follow; therefore, incomplete surgery is responsible for these many failures, and not unnecessary and uncalled for surgery.

As we said before in our paper, the great difficulties in connection with our work is the uncertainty of diagnosis and the varied and complicated and unique expressions, which so many varied and different pathological phenomena can and will produce. The nervous symptoms of retroversions and posterior displacements are much like those of floating-kidney, and if both conditions exist in the same patient, operations for either one trouble, seldom relieve all the symptoms present, and no one can be satisfied beforehand whether both operations will not be necessary to complete a cure.

Thus, the clinical picture of disease in its entirety, must be carefully studied, and each individual element eliminated and given its proportionate consideration, and such remedial measures instituted, as scientific medicine, based upon a broad and enlightened pathology suggests.

493 Delaware Avenue.

